# Transesophageal echocardiographic imaging of multiple complications following mitral valve replacement

**DOI:** 10.1530/ERP-15-0026

**Published:** 2015-10-19

**Authors:** Charles L Brassard, Claudia Viens, André Denault, Pierre Couture

**Affiliations:** 1 Université Laval, Quebec City, Quebec, Canada; 2 Montreal Heart Institute, Université de Montréal, Montreal, Quebec, Canada

## Abstract

We present a case of mitral valve (MV) replacement that resulted in multiple complications, as diagnosed by transesophageal echocardiography (TEE), including left ventricular outflow tract obstruction, aortic dissection and left ventricular rupture. We also describe that identification of bleeding originating from the posterior aspect of the heart by the surgical team should trigger a complete TEE evaluation for adequate diagnosis. An 84-year-old woman underwent a MV replacement. Weaning from cardiopulmonary bypass (CPB) revealed a late-peaking gradient of 44 mmHg over the left ventricular outflow tract caused by obstruction from a bioprosthetic strut. After proper surgical correction, TEE evaluation showed a type A aortic dissection that was subsequently repaired. After separation from CPB, the surgical team identified a major bleed that originated from the posterior aspect of the heart. Although the initial suspicion was injury to the atrioventricular groove, a complete TEE evaluation confirmed a left ventricular free wall rupture by showing the dissecting jet using colour-flow Doppler. TEE is an essential component in cardiac surgery for assessment of surgical repair and potential complications. Posterior bleeding should trigger a complete TEE examination with assessment of nearby structures to rule out a life-threatening pathology. Left ventricular free wall rupture can be identified using colour-flow Doppler.

**Learning points:**

- Multiple complications may occur after MVR.
- TEE is an essential component in the evaluation of surgical repair and its potential associated complications, including LVOT obstruction, aortic dissection and LV rupture.
- Posterior bleeding, from the region of AV groove, should trigger a complete TEE examination with assessment of nearby structures such as the atria, coronary sinus and myocardium to rule out a life threatening pathology.
- The diagnosis of a LV rupture can be confirmed with 2-D imaging and colour-flow Doppler demonstrating a dissecting jet through the myocardium.

## Background

The role of intraoperative transesophageal echocardiography (TEE) has increased tremendously over the years. Today, intraoperative TEE is a class 1 indication for mitral valve (MV) surgery in the evaluation of pathology, surgical repair and associated complications. The authors present a case report highlighting the value of TEE in the assessment of multiple complications following MV replacement surgery including, left ventricular outflow tract obstruction, aortic dissection and left ventricular rupture. Written consent about utilization of patient information and imaging was obtained from the patient during the preoperative evaluation.

## Case presentation

An 84-year-old woman with a diagnosis of severe MV regurgitation was referred to our institution for a MV replacement. Her past medical history included diastolic heart failure, pulmonary hypertension and paroxysmal atrial fibrillation. After uneventful induction of general anaesthesia, transesophageal echocardiographic (TEE) examination demonstrated a small retracted posterior leaflet with absence of coaptation. Additional findings included a hyperdynamic left ventricle (LV) with preserved systolic function and no regional wall motion abnormalities (Video 1). Written consent about the utilization of patient information and imaging was obtained from the patient during the preoperative evaluation.

Video 1 This video demonstrates pre-repair clips and is divided into three sections. The initial images present a mid-esophageal four-chamber view showing a dilated left atrium and absence of mitral leaflet coaptation. The next section demonstrates severe mitral insufficiency by colour-flow Doppler. The last section shows a hyperdynamic left ventricle with absence of pericardial haematoma. View Video 1 at http://movie-usa.glencoesoftware.com/video/10.1530/ERP-15-0026/video-1.
Download Video 1

## Investigation

**Figure 1:**
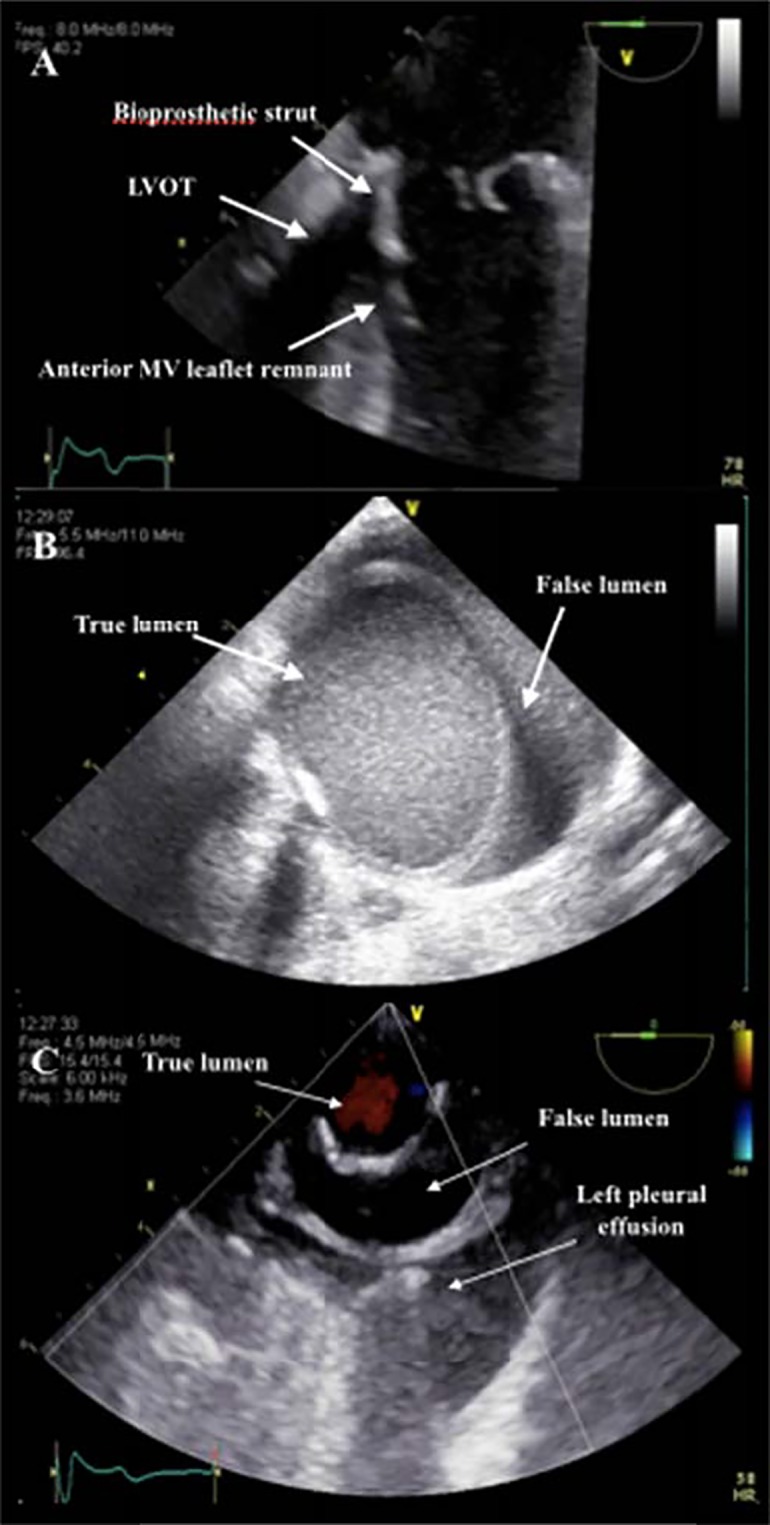
(A) Mid esophageal five-chamber view demonstrating LVOT obstruction from a bioprosthesis strut and anterior leaflet remnants. (B) Epiaortic ultrasound showing dissection of the ascending aorta. (C) Short axis view of the descending aorta with colour-flow Doppler to identify the true lumen of the aortic dissection. LVOT, left ventricular outflow tract; MV, mitral valve.

**Figure 2:**
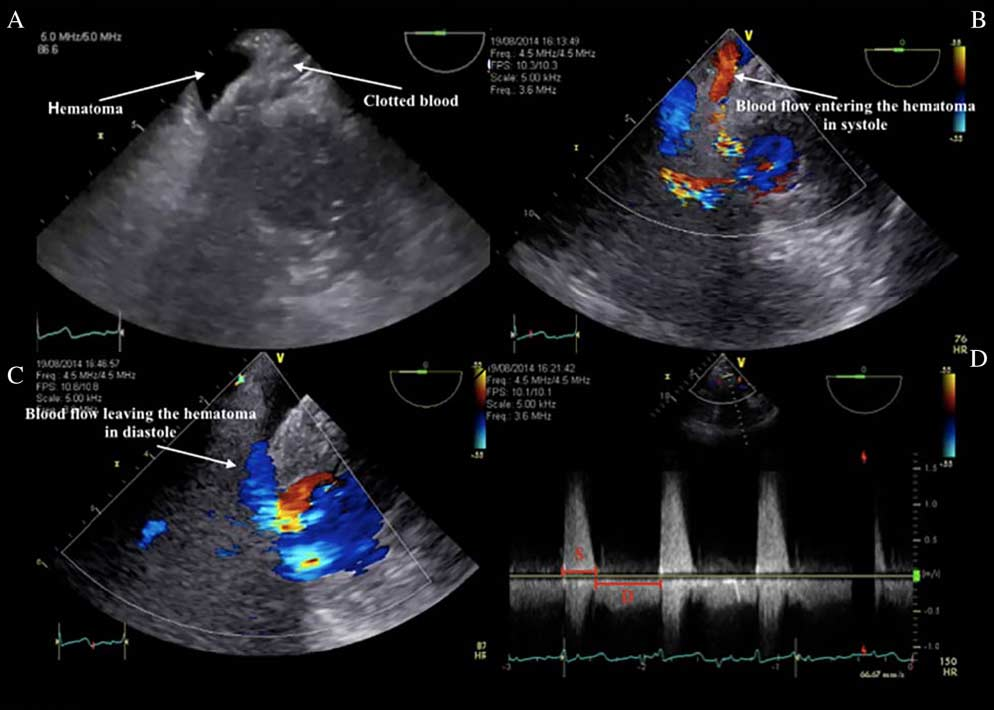
(A) Transgastric mid-papillary views demonstrating a hypoechogenic density, i.e., pericardial haematoma adjacent to clotted blood. (B) CF Doppler demonstrating blood flow entering the haematoma. (C) CF Doppler demonstrating blood flow leaving the haematoma. (D) CW Doppler showing the timing of blood flow through the LV rupture relative to the cardiac cycle. Blood blow is entering the haematoma in systole and leaving the haematoma in diastole. LV, left ventricle; CF, colour-flow; CW, continuous wave; S, systole; D, diastole.

Intraoperative findings showed no valvular calcification and a 29 mm St Jude Medical Epic porcine bioprosthesis was implanted without resection of native leaflets and subvalvular apparatus. As the patient was weaned from cardiopulmonary bypass (CPB), left ventricular outflow tract (LVOT) obstruction was observed. One of the bioprosthetic struts was seen in the LVOT (despite a typical commissural positioning of the prosthesis), in addition to anterior leaflet remnants (Fig. 1A). Continuous-wave (CW) Doppler showed a late-peaking gradient of 44 mmHg over the LVOT. Because of associated hemodynamic instability, CPB was then reinitiated. After removal of the bioprosthesis, the anterior leaflet was completely resected with preservation of papillary muscles, and a new 29 mm St Jude bioprosthesis was inserted. No more LVOT obstruction was observed. However, during the second trial of weaning from CPB, a type A aortic dissection was visualized (Fig. 1B and C). After appropriate cooling, the ascending aorta and hemiarch were replaced using a 28 mm graft. A total circulatory arrest time of 21 min was recorded. Afterwards, separation from CPB was established for a second time. However, significant bleeding originating from the posterior aspect of the heart occurred and was associated with hemodynamic instability. The surgical team suspected a potential injury to the atrioventricular groove (AVG). A retroflexed mid-esophageal four-chamber view revealed a compressed coronary sinus (CS) measured at 5 mm, which prompted further examination of the AVG (Video 2). Ventricular rupture and a pericardial haematoma were visible from the trans-gastric short axis views. Colour-flow (CF) Doppler easily identified a jet dissecting through the posterior ventricular free wall. There was a large amount of blood flow entering the haematoma in systole, and leaving the haematoma in diastole CW Doppler confirmed the pattern of flow respective to the cardiac cycle (Fig. 2 and Video 2).

Video 2 This video demonstrates post-repair clips with the appearance of a newly compressed coronary sinus. The next clip shows a transgastric mid-papillary view with a haematoma in the inferoseptal region of the myocardium with adjacent clotted blood. The last two clips demonstrate the LV rupture with colour-flow Doppler. LV: left ventricle. View Video 2 at http://movie-usa.glencoesoftware.com/video/10.1530/ERP-15-0026/video-2.
Download Video 2

## Treatment and outcome

Considering the prolonged total CPB time of over 6 h, the risk of additional aortic clamping and the age of the patient, the mid-ventricular rupture was repaired off pump. After achieving a reasonable haemostasis, the patient was transferred to the intensive care unit with substantial inotropic support. However, she died in the same evening. Autopsy was not permitted.

## Discussion

The above-mentioned case describes three different complications after MV replacement surgery. TEE plays a central role in the evaluation of surgical repair and potential associated complications.

The decision to return on CPB for a second MV replacement was based on TEE findings of a LVOT obstruction from the strut of the bioprosthetic valve and anterior leaflets remnants. This is a well described phenomenon in the literature (1). Preservation of subvalvular apparatus maintains LV function and reduces the risk of myocardial rupture; however it harbours the potential for LVOT obstruction. Risk factors for the development of LVOT obstruction post MVR have include high profile prosthetic valve, a small LV cavity, left ventricular hypertrophy, redundancy of the chordal apparatus and incorrect positioning of the prosthesis (2, 3).

Intraoperative aortic dissection is a rare but potentially fatal iatrogenic complication of open-heart surgeries. The incidence of intraoperative aortic dissection in cardiac surgical procedures was found to be 0.13–0.35% in several retrospective studies, with a mortality rate ranging from 20% to 33%. Dissections mostly originate from the aortic cannulation, the cross-clamp site or at the site of the partial-occlusion clamp (4). Several TEE standard views can be used to visualize the proximal ascending aorta, aortic arch and descending thoracic aorta. However, shadowing from air-filled trachea and the right main-stem bronchus may obscure the mid and distal ascending aorta where cannulation and cross-clamping are performed. Previous studies indicate that the onset of aortic dissection occurs either shortly after the start of CPB or following termination of the procedure; hence, TEE examination of every segment of the aorta, with colour-flow Doppler, should be routine to enable early diagnosis and treatment.

Rupture of the LV is a rare but often fatal complication after MV replacement. Classification of LV rupture can be categorized into three types based on the anatomical location of the rupture and the mechanism involved. Type 1 is a tear in the AVG associated with extensive decalcification of the MV annulus, resection of posterior leaflet or improper lifting of the heart. Type 2 is a tear at the base of the papillary muscle associated with excessive resection of posterior papillary muscle. Type 3 is a tear located between type 1 and type 2 lesions most often related to trauma of the posterior ventricular wall from a large prosthetic valve and penetration of a valve strut in the posterior myocardium (5).

In our case, bleeding was identified in the mid-ventricular region of the posterior wall. TEE examination confirmed a mid-ventricular rupture near the ventricular septum extending to the LV inferobasal segment with flow entering the pericardial space in systole, as documented by continuous wave Doppler. An echo-lucent haematoma was also visualized adjacent to clotted blood in the posterior region. The most likely etiology of the bleeding was a type 3 LV rupture of the posterior wall by penetration of a valvular strut.

Although bleeding from the region of the AV groove may originate from an AVG dissociation or rupture of the LV free wall, it is important to differentiate both mechanisms from other etiologies such as injury to the CS or left atrium. Haematoma in the AVG from CS trauma has previously been identified as an echo-lucent space adjacent to the left atrial wall with an absence of flow (6). Chui *et al*. (7) recently described an atrioventricular dissociation (type 1 LV rupture) after MV replacement. Initial TEE images revealed a small interatrial haematoma, paravalvular leak, rocking motion of the MVR associated with significant hemodynamic instability and a large posterior AVG haematoma. Because CS compression may also occur with AV dissociation or myocardial rupture, adequate diagnosis is essential as they mandate immediate repair. AVG haematoma, secondary to CS injury may be treated by conservative management, unless associated with hemodynamic instability or pulmonary vein obstruction (6).

## Patient consent

The patient was deceased and therefore could not give consent.

## Author contribution statement

C L Brassard, C Viens and A Denault helped review the original data and write the manuscript. P Couture helped review the original data and write the manuscript and served as the archival author.
